# Confirmed Case of Buruli Ulcer, Senegal, 2018

**DOI:** 10.3201/eid2503.180707

**Published:** 2019-03

**Authors:** Grace Anne Turner, Abdoulave Seck, Assane Dieng, Saër Diadie, Babacar Ndiaye, Tabitha D. van Imeerzeel, Moussa Diallo, Marie Kempf, Raymond Bercion, Cheikh Saad-Bouh Boye

**Affiliations:** Keru Yakaar, Dakar, Senegal (G.A. Turner, T.D. van Imeerzeel);; University of Cheikh Anta Diop, Dakar (A. Seck, A. Dieng, S. Diadie, M. Diallo, C.S.-B. Boye);; Pasteur Institute, Dakar (A. Seck, B. Ndiaye, R. Bercion);; University of Angers, Angers, France (M. Kempf);; University Hospital Center, Angers (M. Kempf); University Hospital Center Dantec, Dakar (C.S.-B. Boye)

**Keywords:** Buruli ulcer, Mycobacterium ulcerans, West Africa, Senegal, risk factors, cutaneous mycobacterium infections, bacteria, tuberculosis and other mycobacteria

## Abstract

Buruli ulcer is a necrotizing skin disease caused by *Mycobacterium ulcerans* and is usually associated with tropical climates and exposure to slow-moving or stagnant water. We report a case of Buruli ulcer that may have originated in an urban semiarid area of Senegal.

In January 2018, a 14-year-old boy came to an urban clinic in Dakar, the capital of Senegal, with a 2-week history of skin lesions. He had a 1 × 1 cm ulcerous erosion over a 6 × 16 cm painful edematous lesion on his right calf; he was febrile, with a temperature of 38.5°C. He was initially treated for cellulitis with amoxicillin and clavulanate acid, along with wound care. Two days later, the lesion had evolved. Debridement revealed considerable necrotic subcutaneous tissue extending 1–3 cm under the epidermal edge. The most proximal of the 3 ulcers had a diameter of 1 cm, the next measured 5 × 6 cm, and the last was an L-shaped lesion measuring 6 × 28 cm, running from midcalf to toes. Infection with *Mycobacterium ulcerans* was suspected because of rapid tissue necrosis, classic undermining edges, patient age, location of the lesions, and failure of standard care (Appendix Figure 1). 

The patient was admitted to the hospital and treated with parenteral gentamicin, oral metronidazole, and wound care. The wound bed was swabbed; culture revealed *Acinetobacter* and *Pseudomonas.* Antimicrobial drug therapy was changed to parenteral gentamicin and oral ciprofloxacin. Four swab specimens were obtained from the wound, and quantitative real-time PCR assay targeting the IS2404 putative transposase gene and the mycolactone polyketide synthase gene confirmed the presence of *M. ulcerans*. Targeting IS2404 is considered the diagnostic standard for Buruli ulcer (*1*). Targeting IS2404 PCR analysis for *M. tuberculosis* and negative controls were both negative (Appendix). A skin graft was performed, and the patient was discharged and given rifampin/isoniazid, ciprofloxacin, and wound care.

The patient had been born in rural Guinea-Conakry and moved to Senegal 3 years before his illness. His mother reported that he had been fully vaccinated, although no records remain. He moved to Senegal in 2015 and lived in Dakar for 18 months, then moved east to the semiarid area of Diourbel to attend Koranic school for another 18 months. He denied engaging in any agricultural or mining activities or bathing, washing, or swimming in bodies of fresh water during his 3 years in Senegal. He also denied returning to Guinea-Conakry or other travel since his arrival in Senegal. In Guinea-Conakry, he had been involved in agricultural activities, including rice farming. The family does not use mosquito nets, and he reported occasional insect bites.

Worldwide, Buruli ulcer is the third most common mycobacterial infection, inflicting debilitating cost and social stigma on patients and their families (*2**,**3*). The highest incidence of Buruli ulcer is found in tropical or subtropical sub-Saharan Africa, but 2 cases have been reported in Mali, a semiarid country not usually associated with Buruli ulcer (*3*–*5*). The only other known case of Buruli ulcer in Senegal was in a traveler from Europe who had been building canoes in fresh water along the tropical Senegal–Guinea border (*6*).

The mode of transmission of *M. ulcerans* is poorly understood and may vary by region. The bacterium has been found in aquatic environments, animals, and insects. Animal reservoirs and insect vectors have been proposed, but no definitive vector has been identified (*7*). A systematic review found that poor wound care, living or working near aquatic environments, and failure to wear protective clothing (long pants and long-sleeved shirts) were risk factors associated with *M. ulcerans* infection. Results among other researchers searching for risk factors have been contradictory (*8*). The reported incubation period ranges between 34 and 264 days, with a mean of 4.5 months (*9*). A multicenter study in West Africa demonstrated no significant evidence of protection from *M. ulcerans* infection after bacillus Calmette-Guérrin vaccination (*10*).

This case of Buruli ulcer is noteworthy because it is a confirmed case originating in a semiarid region of West Africa, suggesting that the endemic area of this disease is poorly defined or changing. The patient appears to have contracted the disease in Senegal without the usual water-related risk factors, although he was exposed to insect bites. It is possible but unlikely that he contracted the disease in Guinea-Conakry 3 years earlier, which would mean that he had an incubation period 2 years longer than any previously reported cases. There is no evidence to suggest his possible bacillus Calmette-Guérrin vaccination delayed wound development.

This case illustrates the need to better define the geographic extent and modes of transmission of this debilitating disease so that primary control measures can be identified. In addition, health workers must be provided with the training and tools to diagnose and treat *M. ulcerans.* Research into a point-of-care diagnostic test is needed so that timely treatment can minimize disability and costs to the family.

Appendix Additional details on confirmed case of Buruli ulcer, Senegal, 2018.
