# Supplementary material for: Confirmed Case of Buruli Ulcer, Senegal, 2018
Source: Emerg Infect Dis. 2019 Mar;25(3):600–1. doi: 10.3201/eid2503.180707 (PMC6390742; doi:10.3201/eid2503.180707)
Supplement: Appendix — Additional details on confirmed case of Buruli ulcer, Senegal, 2018. [file 18-0707-Techapp-s1.pdf]

Article DOI: https://doi.org/10.3201/eid2503.180707

Confirmed Case of Buruli Ulcer, Senegal, 2018

Appendix

Details of PCR Analysis

Materials and Methods

We took a total of 12 swabs, as well as a small piece of biopsied tissue, and homogenized them in sterile water. We divided the swabs into groups of 4 (named "swab 1" to "swab 3"); the small piece of biopsy was named "swab 4." For DNA extraction, we washed 400 µL of the sample 3 times with sterile water then centrifuged it at 15.000 × *g* for 15 min. We resuspended the pellet in 50 µL of NaOH 50 mM and heated it at 95°C for ≥15 min. We then purified the DNA using the QIAquick PCR purification kit (QIAGEN, https://www.qiagen.com/) after adjusting the pH by adding 10 µL of 3 M sodium acetate pH 5.2, following the manufacturer's recommendations.

We determined the presence of *Mycobacterium ulcerans* by quantitative real-time PCR assay targeting the IS2404 putative transposase gene using the primers MuF1: 5′-TTGGTGCCGATCGAGTTG-3′, MuR1: 5′-CGCTTTGGCGCGTAAA-3′ with the dye MuFT1: FAM-CACCACGCAGCATTCTTGCCGT-BHQ1 (*1*), and the mycolactone polyketide synthase gene, using the primers KRTF: 5′- TCACGGCCTGCGATATCA-3′, KRTR 5′-TTGTGTGGGCACTGAATTGAC-3′ and the dye FAM-ACCCCGAAGCACTGGCCGC-BHQ1 (*2*). We performed the amplifications using thermocycler conditions as follows: an initial denaturation step at 95°C for 3 min followed by 40 cycles of denaturation at 95°C for 15 sec and extension at 60°C for 1 min.

We determined the presence of *Mycobacterium tuberculosis* complex by quantitative real-time PCR assay targeting the IS6110 insertion element using the primers Tb762F: 5′-CCTGCGAGCGTAGGCGT –3′, Tb762R: 5′- CTCGTCCAGCGCCGCTT –3′ and the dye

S762FB: FAM- GACAAAGGCCACGTAGGCGAACCCT -BHQ1. We performed the amplification using the following thermocycler conditions: an initial denaturation step at 95°C for 3 min followed by 45 cycles of denaturation at 95°C for 15 sec and extension at 60°C for 20 sec.

For quantification of DNA, we used 10-fold dilutions of known concentration of *M. ulcerans* or *M. tuberculosis* complex positive control. We also included a negative control in each assay.

Results

We observed amplification for the *M. ulcerans* IS2404 gene in the 4 samples, with a quantity of DNA detected ranging from 6.3×10^2 to 4.6×10^3 genome units (GU) per mL (Appendix Table 1 and Appendix Figure 2), and for the mycolactone polyketide synthase gene, with a quantity of DNA detected ranging from 4.3×10^2 to 1.6×10^4 GU per mL (Appendix Table 2). No DNA amplification was observed for the *M. tuberculosis* complex IS6110 gene or for the negative controls.

References

1. Rondini S, Mensah-Quainoo E, Troll H, Bodmer T, Pluschke G. Development and application of real-time PCR assay for quantification of *Mycobacterium ulcerans* DNA. J Clin Microbiol. 2003;41:4231–7. http://dx.doi.org/10.1128/JCM.41.9.4231-4237.2003

2. Fyfe JA, Lavender CJ, Johnson PD, Globan M, Sievers A, Azuolas J, et al. Development and application of two multiplex real-time PCR assays for the detection of *Mycobacterium ulcerans* in clinical and environmental samples. Appl Environ Microbiol. 2007;73:4733–40. http://dx.doi.org/10.1128/AEM.02971-06

Appendix Table 1. Cycle threshold (C_t) value of PCR targeting *M. ulcerans* IS2404 and mean quantity of genome unit per mL obtained for each swab.

Sample	C_t value of PCR targeting IS2404 (2 points performed)	Mean quantity of genome unit per mL
Positive control dilution 10^{-1}	30.09, 20.08	3.50×10^6
Positive control dilution 10^{-2}	23.55, 23.55	3.50×10^5
Positive control dilution 10^{-3}	26.99, 26.74	3.50×10^4
Positive control dilution 10^{-4}	29.97, 30.12	3.50×10^3
Positive control dilution 10^{-5}	34.11, 34.02	3.50×10^2
Negative control	No C_t	0
Swab 1	29.98, 29.96	4.56×10^3
Swab 2	30.13, 29.91	4.43×10^3
Swab 3	32.94, 32.45	7.49×10^2
Swab 4	32.19, 32.30	1.00×10^3

Appendix Table 2. Cycle threshold (C_t) value of PCR targeting mycolactone polyketide synthase gene obtained for each swab*

Sample	C_t value of PCR targeting mycolactone polyketide synthase gene (2 points performed for controls and 6 for clinical samples)
Positive control dilution 10^{-1}	23.61, 23.42
Positive control dilution 10^{-2}	27.32, 27.16
Positive control dilution 10^{-3}	30.60, 30.71
Positive control dilution 10^{-4}	35.01, 33.62
Positive control dilution 10^{-5}	36.61, 37.65
Negative control	No C_t, No C_t
Swab 1	No C_t (×5), 37.14
Swab 2	No C_t (×3), 35.54, 35.57, 35.68
Swab 3	No C_t (×3) 34.62, 33.33, 34.03
Swab 4	32.06, 31.53, 31.92, 31.29, 31.71, 32.26

*For quantification of DNA, 10-fold dilutions of known concentration of *M. ulcerans* were tested (positive control dilution 10^{-1} to 10^{-5}). The negative control corresponds to water rather than DNA extract.

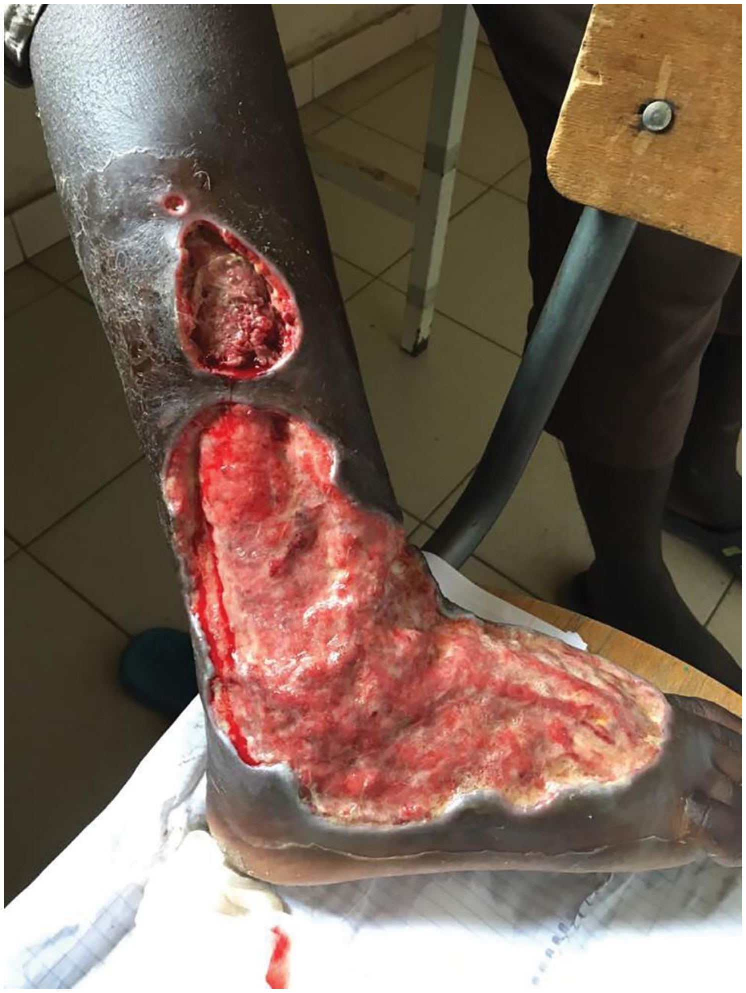

Appendix Figure 1. Leg infected with *Mycobacterium ulcerans* (Buruli ulcer) with undermining edges.

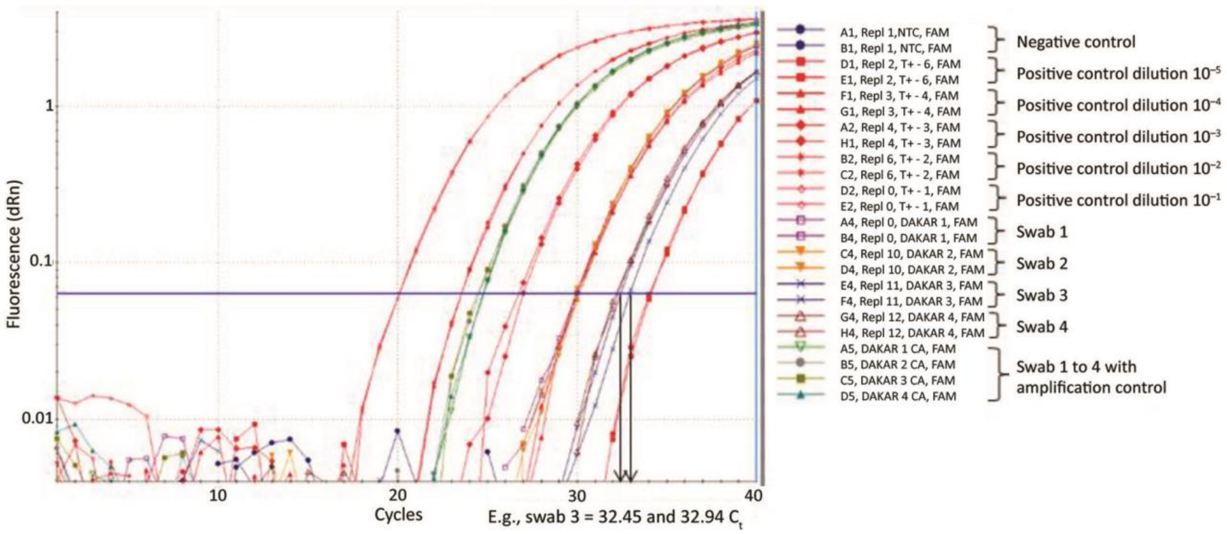

Appendix Figure 2. Amplification plots of *Mycobacterium ulcerans* IS2404 gene.
